# Supplementary material for: Mutations in the kinesin KIF12 promote MASH in humans and mice by disrupting lipogenic enzyme turnover
Source: EMBO J. 2025 Feb 7;44(6):1608–40. doi: 10.1038/s44318-025-00366-8 (PMC11914266; doi:10.1038/s44318-025-00366-8)
Supplement: Supplementary file 1 — Table EV1 [file 44318_2025_366_MOESM1_ESM.docx]

Table EV1. Sequences of the synthesized oligonucleotides, corresponding to Fig. 6A.

| Name | Sequence |
| --- | --- |
| *K12 Head F* | 5’-TTAAAGCTTCCATGGAGGAACGTGGGTCTC-3’ |
| *K12 Head R* | 5’-ATATATGTCGACGGTGGCTTTACTCCAGGG-3’ |
| *K12 Stalk F* | 5’-TTGTCTCGAGCCATGGCGCAGCAAGTAGAGAACGAGTTG-3’ |
| *K12 Stalk R* | 5’-TAATGTCGACAAAAGGAGGCGCCTCTCCAG-3’ |
| *K12 PRD F* | 5’-TTTTCTCGAGCCATGGCGCCCCTTCCCCAGCAGG-3’ |
| *K12 PRD R* | 5’-ATTTGTCGACAATGGGGGCTTGGCAGAGC-3’ |
| *K12 Tail F* | 5’-TTTTCTCGAGCCATGGCGTCGGCTTGTCCCCTTCC-3’ |
| *K12 Tail R* | 5’-ATGTCGACAAACAAGAAGAGAGAGGCCTTGG-3’ |
| *N-BAM-ATG* | 5’-ACTGGATCCATGGGACCCCTTCCCCAGCAGG-3’ |
| *C-BAM-NoStop* | 5’-ACTGGATCCTGGGGGCTTGGCAGAGCCTG-3’ |
| *Mut1-1* | 5’-CAGGCAACCGTGAGTGTGACCCCACGG-3’ |
| *Mut1-2* | 5’-CCGTGGGGTCACACTCACGGTTCCTGCC-3’ |
| *Mut2-1* | 5’-GCGGATCACCACCTGACCACAGGGTCCCA-3’ |
| *Mut2-2* | 5’-TGGGACCCTGTGGTCAGGTGGTGATCCGC-3’ |
| *Clinical-Mut-seq* | 5’-GCCAATAGCATCAACCGCAG-3’ |
| *ACC-GFP-F* | 5’-ACTGAGCTCCATGGATGAACCATCTCCCTTGG-3’ |
| *ACC-GFP-R* | 5’-ACTCCCGGGACGTGGAAGGGGAAT CCATTG-3’ |
| *GFP-ACC-F* | 5’-ACTGAGCTCAAATGGATGAACCATCTCCCTTGG-3’ |
| *GFP-ACC-R* | 5’-ACTCCCGGGTCACGTGGAAGGGGA ATCCATTG-3’ |
| *ACC1-qpcr-F* | 5’-TCCACTTGGCTGAGCGATTG-3’ |
| *ACC1-qpcr-R* | 5’-CAAGTCAGCAAACTGCACGG-3’ |
| *PC-GFP-F* | 5’-ACTGAATTCAATGCTGAAGTTCCGAACAGT-3’ |
| *PC-GFP-R* | 5’-ACTGGATCCTCATCACTCGATCTCCAGGATGAG-3’ |
| *GFP-PC-F* | 5’-ACTGAATTCCATGGTGAAGTTCCGAACAG-3’ |
| *GFP-PC-R* | 5’-ACTGGATCCTCGATCTCCAGGATGAG-3’ |
| *PC-qpcr-F* | 5’-CCAGAGGCAGGTCTTCTTTG-3′ |
| *PC-qpcr-R* | 5’-GGCCCTTCACGTCCTTTAG-3 |
| *KIF12-qpcr-F* | 5’-GACCAAATGGACTGCAAGGC-3’ |
| *KIF12-qpcr-R* | 5’-CCAGGTCTCGGCTATTCTGC-3’ |
| *GAPDH-qpcr-F* | 5’-CCCACTCCTCCACCTTTGAC-3’ |
| *GAPDH-qpcr-R* | 5’-CCAGGTCTCGGCTATTCTGC-3’ |
