# Supplementary material for: Mutations in the kinesin KIF12 promote MASH in humans and mice by disrupting lipogenic enzyme turnover
Source: EMBO J. 2025 Feb 7;44(6):1608–40. doi: 10.1038/s44318-025-00366-8 (PMC11914266; doi:10.1038/s44318-025-00366-8)
Supplement: Supplementary file 2 — Table EV2 [file 44318_2025_366_MOESM2_ESM.docx]

Table EV2. Summary of *Kif12* gene expression studies on MASH models from public database. Corresponds to Figs. 8E,F and EV7.

| Exp  No. | GEO Acc. No.; Citation | Organ-ism | Strain | Sex | Age | MASH Diet (Model) | Control Diet | MASH Average | Control Average | *p* Va-lues |
| --- | --- | --- | --- | --- | --- | --- | --- | --- | --- | --- |
| 1 | GSE199443 (Boycott *et al*, 2022) | Rat | Fischer 344 (F344) | / | 52 w.o. | CDAA | CSAA | **15.4306** | 0.490753 | 0.00005 |
| 2 | GSE134715 (Boycott *et al.*, 2022) | Rat | Wistar | Male | 18 w.o. | CDDA_Chol_1pc 12 w | CSAA,12 w | **18.5795** | 0.823875 | <0.0001 |
| 3 | GSE134715 (Boycott *et al.*, 2022) | Rat | Wistar | Male | 10 w.o. | CDDA_Chol_1pc 4 w | CSAA 4 w | **3.15225** | 0.2065 | 0.0005 |
| 4 | GSE134715 (Boycott *et al.*, 2022) | Rat | Wistar | Male | 14 w.o. | CDDA_Chol_1pc 8 w | CSAA 8 w | **6.521125** | 0.82325 | <0.0001 |
| 5 | GSE263875 | Mouse | C57BL/6 | Male | / | db/db | WT | 0.123 | **0.131** | 0.4575 |
| 6 | GSE225560 | Mouse | C57BL/6 | / | / | HFD | ND | 11.667 | **13.333** | 0.3753 |
| 7 | GSE274914 (Yadav *et al*, 2024) | Mouse | C57BL/6J | Male | 17 w.o. | HFD 10 w | LFD 10 w | **24.75** | 22.5 | 0.2712 |
| 8 | GSE274914 (Yadav *et al.*, 2024) | Mouse | C57BL/6J | Male | 62 w.o. | HFD 10 w | LFD 10 w | **30.75** | 17 | 0.0663 |
| 9 | GSE274914 (Yadav *et al.*, 2024) | Mouse | C57BL/6J | Fe-male | 17 w.o. | HFD 10 w | LFD 10 w | **29.5** | 24.5 | 0.266 |
| 10 | GSE274914 (Yadav *et al.*, 2024) | Mouse | C57BL/6J | Fe-male | 62 w.o. | HFD 10 w | LFD 10 w | 25.25 | **27** | 0.4059 |
| 11 | GSE246221 | Mouse | C57BL/6J | Male | 20 w.o. | HFD 12 w | SCD 12 w | 19.8 | **25.5** | 0.2485 |
| 12 | GSE273292 | Mouse | C57BL/6 | / | / | HFD 12 w | Chow Diet | 0.04573482 | **0.06095859** | 0.2217 |
| 13 | GSE188128 | Mouse | C57BL/6J | Male | 22 w.o. | HFD 14 w | NFD 14 w | 0.125 | **0.205** | 0.0839 |
| 14 | GSE77625(Soltis *et al*, 2017) | Mouse | C57BL/6J | Male | 24 w.o. | HFD 16 w | Chow Diet | **27.58** | 19.28 | 0.0612 |
| 15 | GSE188344 (Matsushita *et al*, 2022) | Mouse | C57BL/6N | Male | 24 w.o. | HFD 18 w | NCD 18 w | **8.4375** | 5.125 | 0.215 |
| 16 | GSE246221  (Jeong *et al*, 2024) | Mouse | C57BL/6J | Male | 32 w.o. | HFD 24 w | SCD 24 w | **6.333** | 1.5 | 0.085 |

CDAA: Choline-Deficient L-Amino Acid-Defined Diet

CSAA: Choline-Supplemented L-Amino Acid-Defined Control Diet

CDAA_Chol_1pc: Choline-Deficient L-Amino Acid-Defined Diet Supplemented with 1% Cholesterol

HFD: High Fat Diet

ND: Normal Chow Diet

LFD: Low Fat Diet

SCD: Standard Chow Diet

NFD: Normal Fat Diet

NCD: Low-Fat Diet

Bold values: The larger ones.
