# Supplementary material for: Mutations in the kinesin KIF12 promote MASH in humans and mice by disrupting lipogenic enzyme turnover
Source: EMBO J. 2025 Feb 7;44(6):1608–40. doi: 10.1038/s44318-025-00366-8 (PMC11914266; doi:10.1038/s44318-025-00366-8)
Supplement: Supplementary file 4 — Expanded View Figures [file 44318_2025_366_MOESM4_ESM.pdf]

Expanded View Figures

Figure EV1. Symptoms of human pedigrees with KIF12 mutations.

(**A**) MRI images of Patient 2 indicating hepatosplenomegaly. Corresponds to Fig. 1D. (**B**) Summary of blood biochemistry of Patients 2 (Blue) and 3 (Red). Glu glucose, CRE creatinine, TP total protein, ALB albumin, γ-GTP gamma-glutamyl transpeptidase, T-BIL total bilirubin, D-BIL direct bilirubin, LDH lactate dehydrogenase, UR_AC uric acid, CHOL cholesterol. Corresponds to Fig. 1F–H. (**C**) Pedigree of Patient 3's family. Corresponds to Fig. 1F–H.

 © The Author(s)

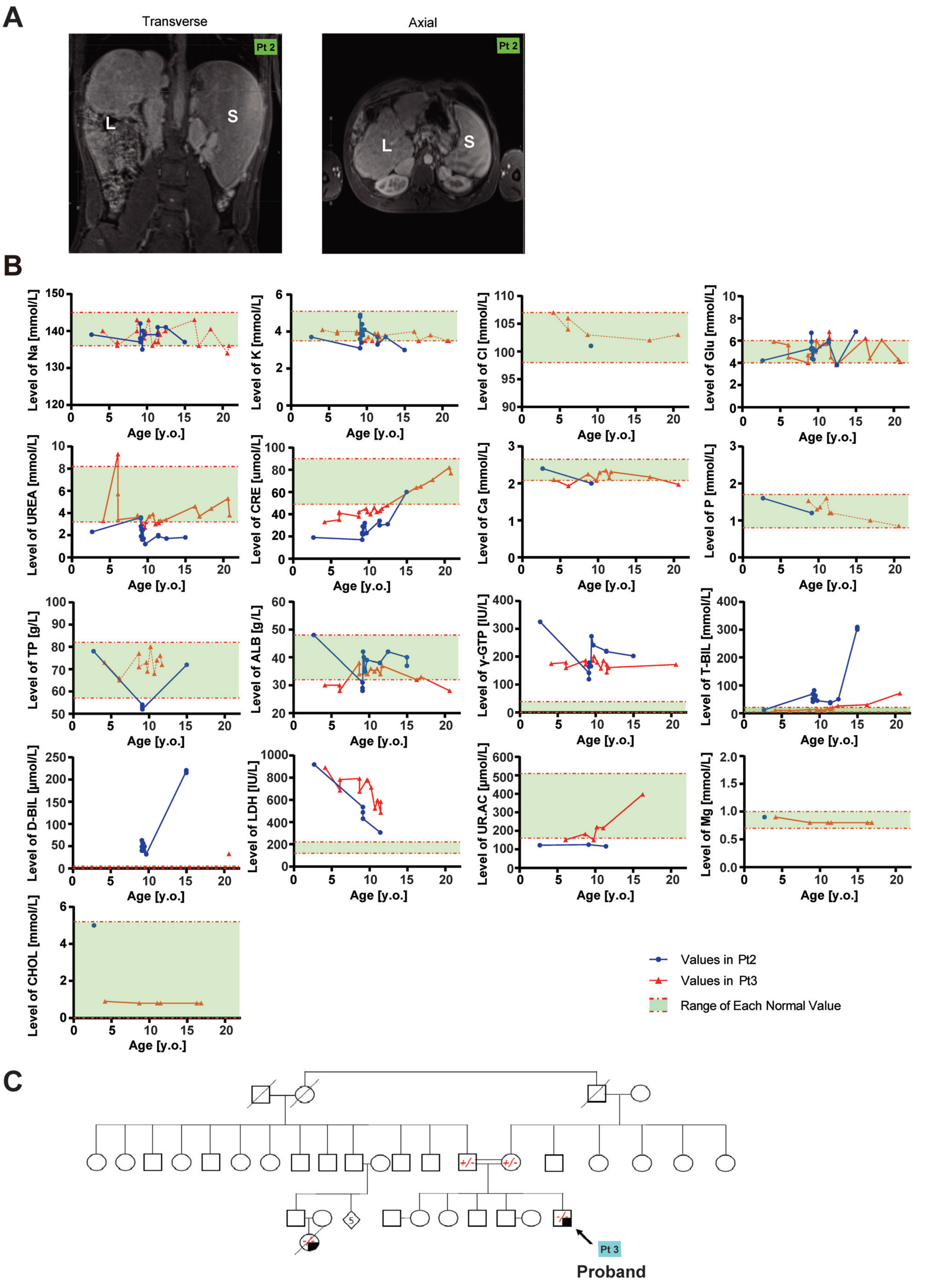
A
Transverse
Axial
Pt 2
L
S
B
Level of Na [mmol/L]
Level of K [mmol/L]
Level of Cl [mmol/L]
Level of Glu [mmol/L]
Level of UREA [mmol/L]
Level of CRE [umol/L]
Level of Ca [mmol/L]
Level of P [mmol/L]
Level of TP [g/L]
Level of ALB [g/L]
Level of γ-GTP [IU/L]
Level of T-BIL [mmol/L]
Level of D-BIL [μmol/L]
Level of LDH [IU/L]
Level of UR.AC [μmol/L]
Level of Mg [mmol/L]
Level of CHOL [mmol/L]
Age [y.o.]
Values in Pt2
Values in Pt3
Range of Each Normal Value
C
+/-
5
Pt 3
Proband

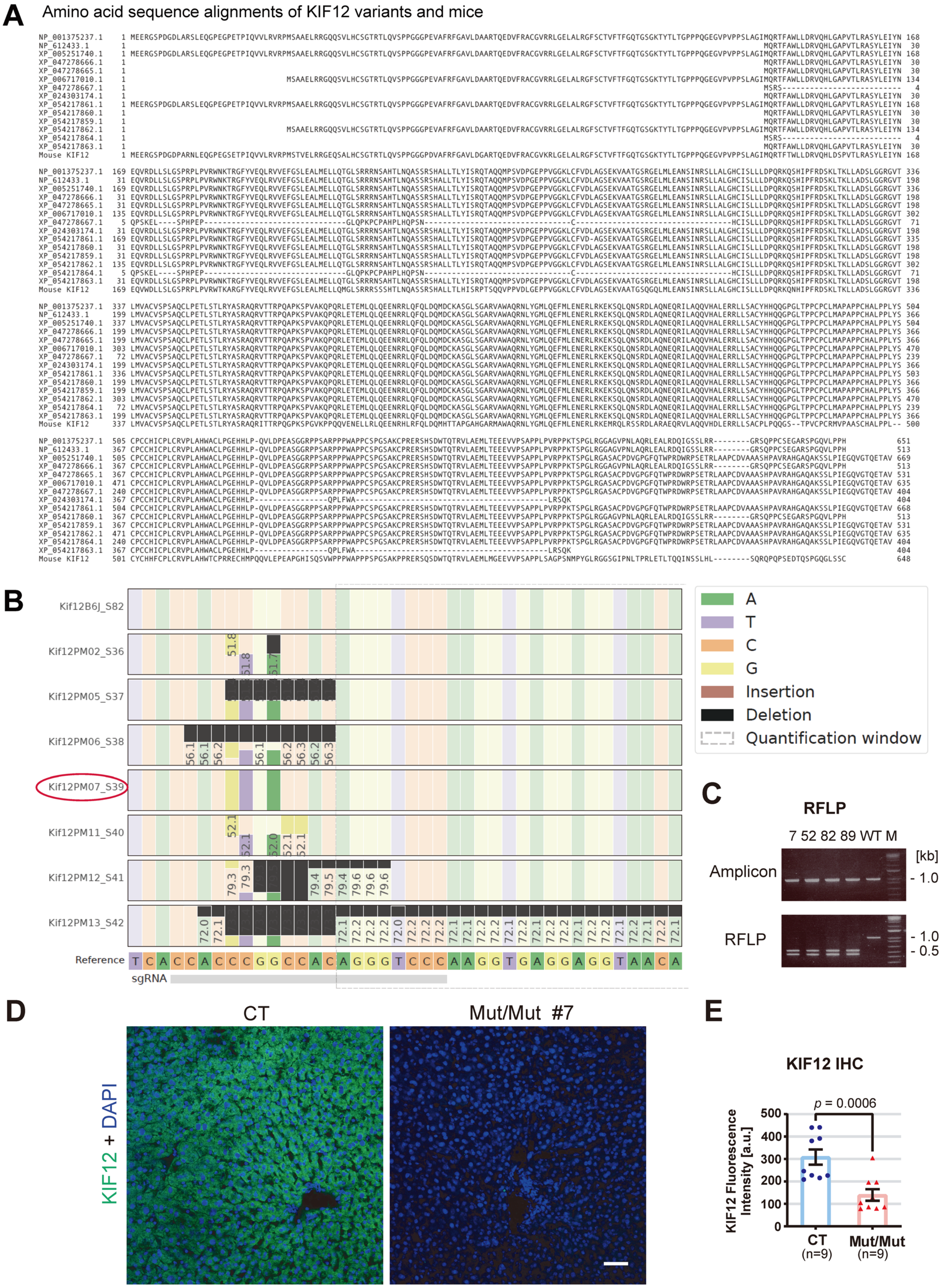
A
Amino acid sequence alignments of KIF12 variants and mice
NP_001375237.1
NP_612433.1
XP_005251740.1
XP_047278666.1
XP_047278665.1
XP_006717010.1
XP_047278667.1
XP_024303174.1
XP_054217861.1
XP_054217860.1
XP_054217859.1
XP_054217862.1
XP_054217864.1
XP_054217863.1
Mouse KIF12
B
Kif12B6J_S82
Kif12PM02_S36
Kif12PM05_S37
Kif12PM06_S38
Kif12PM07_S39
Kif12PM11_S40
Kif12PM12_S41
Kif12PM13_S42
Reference
T C A C C A C C C G G C C A C A G G G T C C C A A G G T G A G G A G G T A A C A
sgRNA
A
T
C
G
Insertion
Deletion
Quantification window
C
RFLP
7 52 82 89 WT M
Amplicon
RFLP
[kb]
- 1.0
- 1.0
- 0.5
D
CT
Mut/Mut #7
KIF12 + DAPI
E
KIF12 IHC
p = 0.0006
KIF12 Fluorescence Intensity [a.u.]
500
400
300
200
100
0
CT
(n=9)
Mut/Mut
(n=9)

 © The Author(s)

Figure EV2. Generation of KIF12 mouse model.

(**A**) Amino acid sequence alignments of human KIF12 variants and a mouse KIF12 sequence (Yang et al, 2014). Corresponds to Fig. 2A. (**B**) Next-generation sequencing of the F0 pups. KIF12PM07_S39 (#7) was turned out to be the correct one. Corresponds to Fig. 2B,C. (**C**) RFLP analysis by *Pml1* restriction digestion. WT, wild type. M, molecular weight markers. Corresponds to Fig. 2B,C. (**D**, **E**) Immunohistochemistry of F0 $Kif12^{mut/mut}$ mouse liver indicating a significant decrease in KIF12 immunofluorescence in the liver of a strain #7 mouse (**D**), accompanied by quantification (**E**). Scale bar, 50 μm. Error bars, mean ± SEM. Welch's *t* test. Biological replicates, 9 optical fields from 3 independent F0 individuals. Corresponds to Fig. 2B,C.

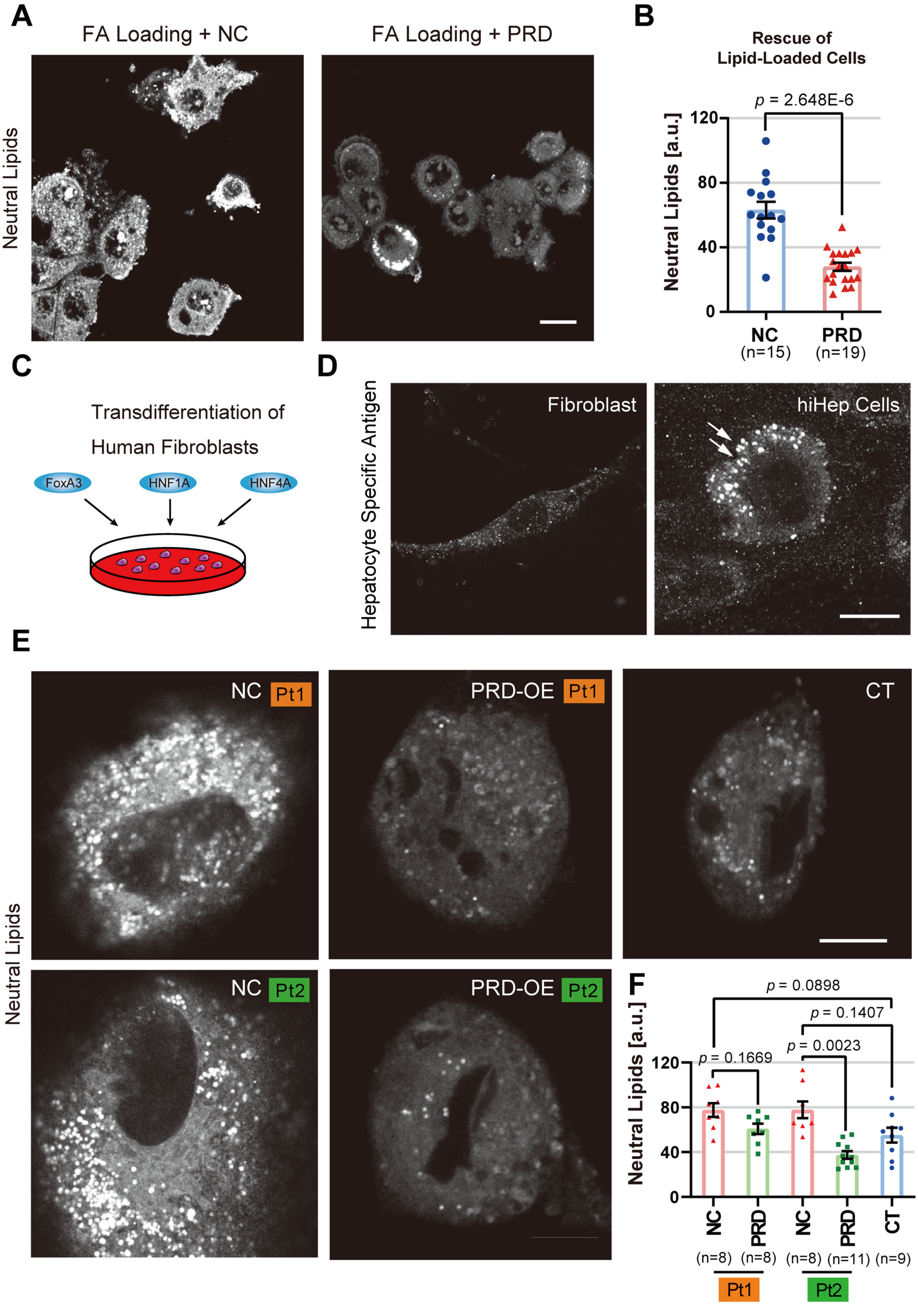
A
FA Loading + NC
FA Loading + PRD
Neutral Lipids
B
Rescue of
Lipid-Loaded Cells
p = 2.648E-6
Neutral Lipids [a.u.]
120
80
40
0
NC
(n=15)
PRD
(n=19)
C
Transdifferentiation of
Human Fibroblasts
FoxA3
HNF1A
HNF4A
D
Hepatocyte Specific Antigen
Fibroblast
hiHep Cells
E
NC Pt1
PRD-OE Pt1
CT
NC Pt2
PRD-OE Pt2
Neutral Lipids
F
p = 0.0898
p = 0.1407
p = 0.0023
p = 0.1669
Neutral Lipids [a.u.]
120
80
40
0
NC
PRD
NC
PRD
CT
(n=8)
(n=8)
(n=8)
(n=11)
(n=9)
Pt1
Pt2

 © The Author(s)

◀ **Figure EV3. KIF12-PRD overexpression ameliorates lipid accumulation in general, corresponding to Fig. 3.**

(**A**, **B**) LipidTOX staining of U0126/oleic-acid-treated (FA loading) HepG2 cells transduced with the mCit-alone (negative control; NC) and mCit-PRD (**A**) and its quantification (**B**). Error bars, mean ± SEM. Welch's *t* test. Biological replicates, individual cells. (**C**, **D**) hiHep cell transdifferentiation from human fibroblasts, indicated by an experimental scheme (**C**) and immunofluorescence microscopy against Hepatocyte-Specific Antigen (HSA) immunocytochemistry (**D**). Scale bar, 10 µm. Arrows in (**D**), HSA signals. (**E**, **F**) LipidTOX neutral lipid staining of patients' (Pt1 and Pt2) and control (CT) hiHep cells without (NC) and with KIF12-PRD overexpression (PRD; **E**), accompanied by statistics (**F**). Scale bar, 10 µm. Error bars, mean ± SEM. One-way ANOVA. Biological replicates, individual cells.

© The Author(s)

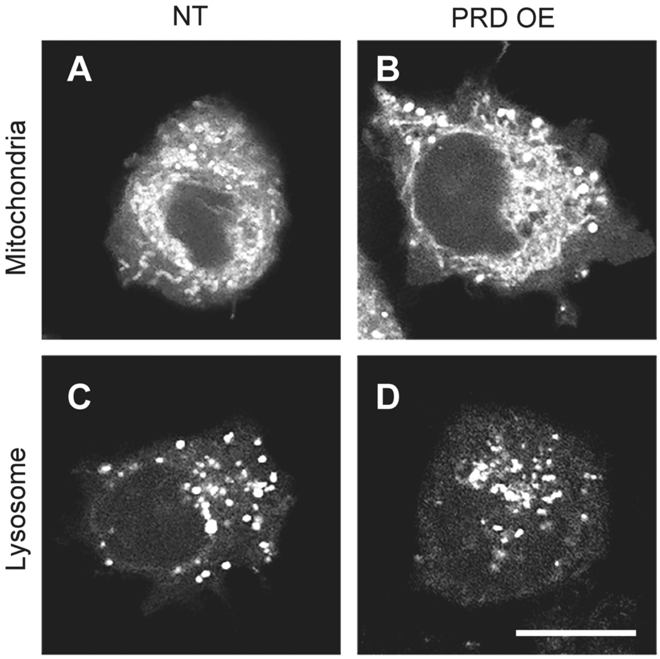

Figure EV4. The effect of PRD overexpression on mitochondrial morphology, corresponding to Fig. 3.

(**A**, **B**) The morphology of mitochondria (**A**, **B**) and lysosomes (**C**, **D**) of HepG2 cells; without (**A**, **C**) or with (**B**, **D**) mCit-PRD overexpression. Note that PRD overexpression tended to increase mitochondrial complexity. Scale bar, 20 µm.

 © The Author(s)

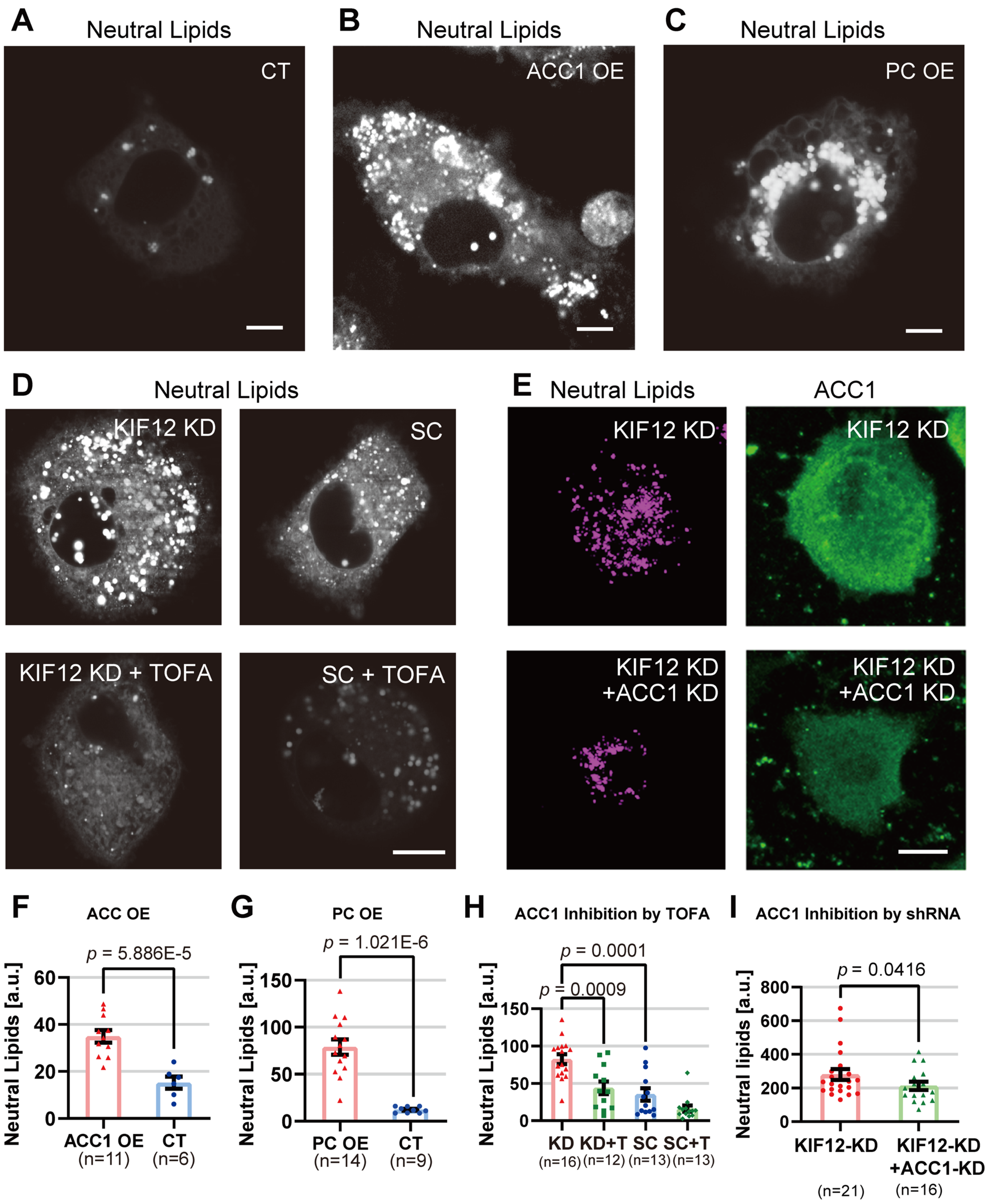
A
Neutral Lipids
CT
B
Neutral Lipids
ACC1 OE
C
Neutral Lipids
PC OE
D
Neutral Lipids
KIF12 KD
SC
KIF12 KD + TOFA
SC + TOFA
E
Neutral Lipids
ACC1
KIF12 KD
KIF12 KD
KIF12 KD +ACC1 KD
KIF12 KD +ACC1 KD
F
ACC OE
p = 5.886E-5
Neutral Lipids [a.u.]
ACC1 OE (n=11)
CT (n=6)
G
PC OE
p = 1.021E-6
Neutral Lipids [a.u.]
PC OE (n=14)
CT (n=9)
H
ACC1 Inhibition by TOFA
p = 0.0001
p = 0.0009
Neutral Lipids [a.u.]
KD (n=16)
KD+T (n=12)
SC (n=13)
SC+T (n=13)
I
ACC1 Inhibition by shRNA
p = 0.0416
Neutral lipids [a.u.]
KIF12-KD (n=21)
KIF12-KD +ACC1-KD (n=16)

Figure EV5. The relevance of ACC1 upregulation in lipid accumulation in KIF12-deficient cells, corresponding to Fig. 5.

(**A–C**) LipidTOX neutral lipid staining of HepG2 cells overexpressing EGFP alone (CT; **A**), ACC1-EGFP (**B**), and PC-EGFP (**C**). Scale bars, 10 μm. (**D**, **E**) LipidTOX neutral lipid staining (**D**, **E**) and ACC1 immunofluorescence (**E**) of HepG2 cells treated by KIF12-KD or SC miRNAs; together with the ACC1 inhibitor, TOFA (2 μg/mL for 24 h; **D**), or with ACC1-KD shRNA (**E**). Scale bars, 20 μm. Repeated twice. (**F–I**) Statistics of neutral lipid staining levels (**F** and **G** for **A–C**; **H** for **D**; **I** for **E**). Error bars, mean ± SEM. One-sided nonpaired Welch's *t* test (**F**, **G**), one-way ANOVA (**H**), and one-sided Mann–Whitney's test (**I**). Biological replicates, individual cells. T, TOFA in (**H**).

 © The Author(s)

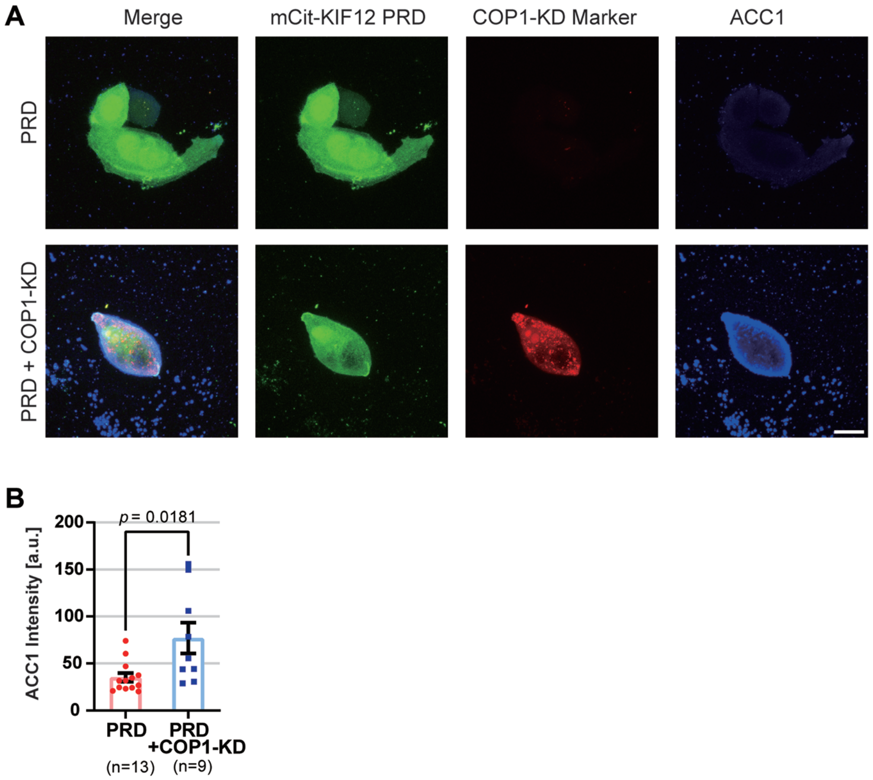

Figure EV6. The relevance of COP1 in ACC1 turnover against lipidosis, corresponding to Fig. 6.

(**A**) ACC1 immunofluorescence with transfection markers of mCit-PRD-overexpressing HepG2 cells, without (PRD) or with treatment by COP1-KD shRNA (PRD + COP1-KD). Scale bars, 20 μm. Note that COP1 deficiency increased the ACC1 level. (**B**) Statistics of (**A**). Error bars, mean ± SEM. One-sided nonpaired Welch's *t* test. Biological replicates, mouse individuals. Corresponds to Fig. 6H,I.

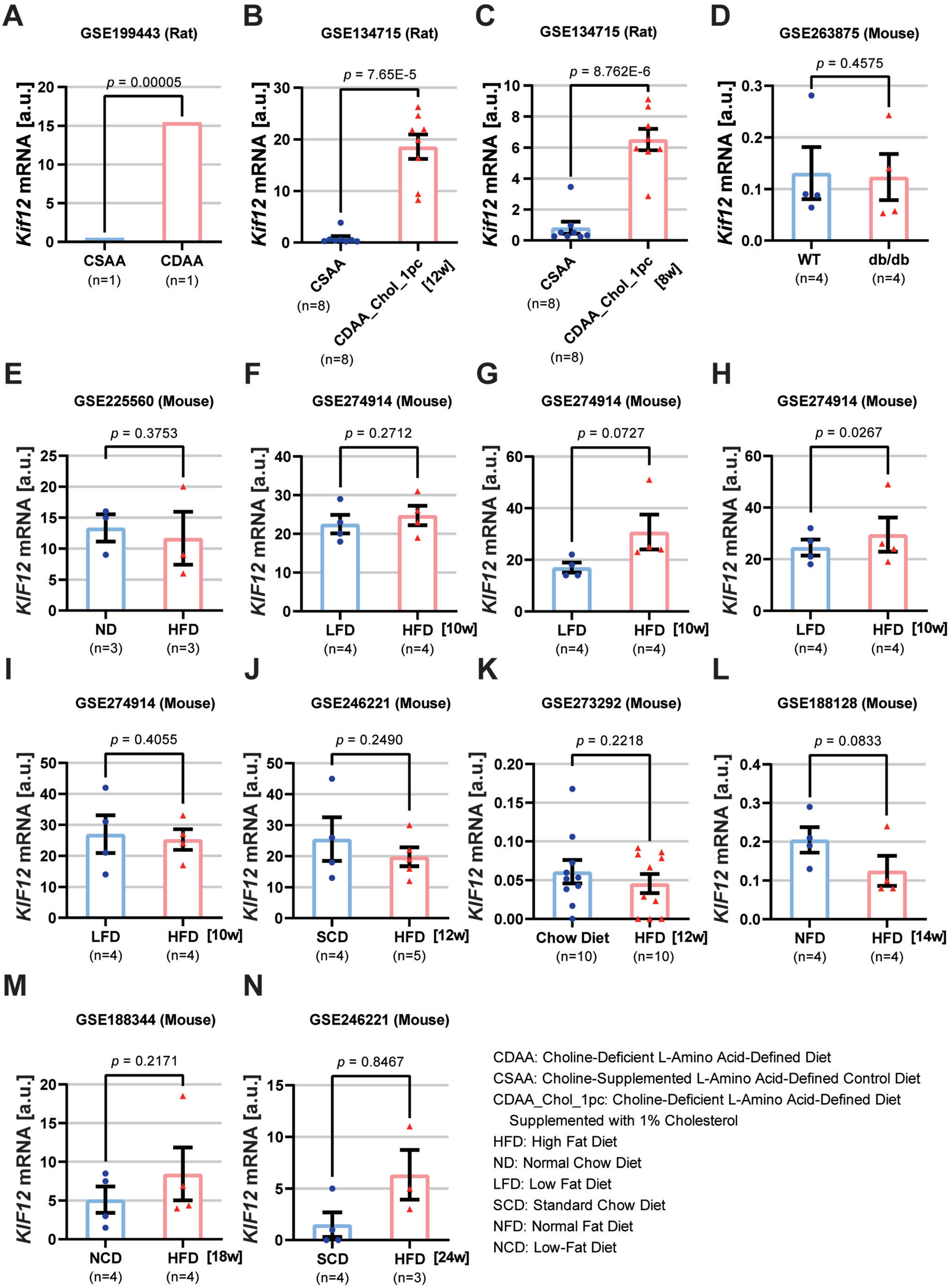

CDAA: Choline-Deficient L-Amino Acid-Defined Diet
CSAA: Choline-Supplemented L-Amino Acid-Defined Control Diet
CDAA_Chol_1pc: Choline-Deficient L-Amino Acid-Defined Diet Supplemented with 1% Cholesterol
HFD: High Fat Diet
ND: Normal Chow Diet
LFD: Low Fat Diet
SCD: Standard Chow Diet
NFD: Normal Fat Diet
NCD: Low-Fat Diet

© The Author(s)

Figure EV7. Summary of *Kif12* transcriptome analyses on MASH rodent models.

(**A–N**) Normalized *Kif12* RNA sequence read number comparison of in vivo samples from rodent MASH models (**A–C**, rats fed with choline-deficient diet; **D**, *db/db* mouse; **E–N**, high-fat diet-fed mice for the indicated periods), according to the indicated public database accession numbers. Error bars, mean ± SEM. Welch's *t* test. Corresponds to Table EV2 and Fig. 8E,F.
